# The Mediating Role of Job Satisfaction in the Relationship between Emotional Intelligence and Life Satisfaction among Teachers during the COVID-19 Pandemic

**DOI:** 10.3390/ejihpe12070050

**Published:** 2022-06-21

**Authors:** Aleksandra M. Rogowska, Hanna Meres

**Affiliations:** Institute of Psychology, University of Opole, 45-040 Opole, Poland; hania.meres@gmail.com

**Keywords:** emotional intelligence, job satisfaction, life satisfaction, teachers, web-based distance education

## Abstract

This study examines the indirect effect of job satisfaction on the relationship between emotional intelligence and life satisfaction among teachers during the second wave of the COVID-19 pandemic in Poland. A sample of 322 teachers aged 23–71 (*M* = 45.37, *SD* = 8.99) participated in a cross-sectional online survey. The online survey (Google form) contained some demographic information and standardized psychological questionnaires: the Multivariate Emotional Intelligence Scale (MEIS) for measuring emotional intelligence, the Minnesota Satisfaction Questionnaire (MSQ)—a short form for job satisfaction assessment, and the Life Satisfaction Scale (SWLS). Emotional intelligence is a significant positive predictor of job satisfaction and life satisfaction, and job satisfaction is a strong positive predictor of life satisfaction. Job satisfaction partly mediates the relationship between emotional intelligence and life satisfaction. To maintain the well-being of teachers during a pandemic, schools should implement training to improve emotional intelligence and increase job satisfaction by supporting distance e-learning among teachers.

## 1. Introduction

The coronavirus disease was first reported in 2019 (COVID-19) in China and spread globally forcing numerous behavioral changes and social isolation. Most schools were temporarily closed during the lockdown in the successive waves of the COVID-19 pandemic [1]. A rapid change in the education system, from the face-to-face classroom to web-based distance learning, was challenging for most teachers and their students [2,3,4,5,6]. The dynamic changes and transformation process required adaptation and resilience skills which relied mainly on the acquisition of new teaching practices and strategies as well as on learning new technological tools and applications compensating for previous face-to-face activities [7,8,9,10,11,12]. However, adjustment to a unique situation was experienced as highly stressful [13,14], significantly contributing to higher rates of anxiety and depression and lower quality of work and life [15,16,17]. García-González et al. [18] found such risk factors for decreased well-being as time pressure, the lack of a schedule, mental overload, and emotional exhaustion. In addition, balancing work and family domains was experienced as very demanding for teachers during the lockdown and significantly impacted their satisfaction with life [19].

One of the resources that can protect against teachers’ mental health deterioration during the COVID-19 pandemic is emotional intelligence [20]. A high level of emotional intelligence helps us recognize, express, understand, and manage positive and negative emotions about ourselves and other people. A significant positive relationship between emotional intelligence and job satisfaction was found in previous studies among teachers [21,22], secondary school heads [23], health care workers [24,25,26], public service workers [27], state civil servants [28], and police officers [29]. Both emotional intelligence and job satisfaction predicted positive organizational commitment [21,29,30]. Furthermore, a mediating effect of job satisfaction on the association between emotional intelligence and organizational commitment was found among nurses [24]. 

Emotional intelligence was also found as a positive predictor of life satisfaction in adolescents [31], undergraduates [32,33,34,35,36,37,38], teachers [39,40,41], and adults from China [42] and Australia [43]. Life satisfaction is one of three core components of subjective well-being and positive and negative affect [44]. Szczygieł and Mikolajczak [38] suggested that the trait of emotional intelligence plays a crucial role in promoting subjective well-being, and its beneficial effect is partially mediated via positive emotion regulation. On the other hand, low emotional intelligence is a predictor of high stress, anxiety, and burnout [34,36,45].

Understanding job satisfaction as a positive cognitive and emotional attitude toward one’s own work may be considered one of several components of the global dimension of life satisfaction [44]. Indeed, studies indicate that job satisfaction, measured in various tools, is strongly and positively related to a high quality of life and satisfaction with life among teachers [39,46] and working adults from Chile [47], China [48], Germany [49], Poland [50], the United States of America [51,52], and citizens of 28 countries from the European Union [53]. Both cross-sectional and longitudinal studies consistently demonstrate that higher job satisfaction predicted higher life satisfaction. Moreover, longitudinal studies indicated that life satisfaction and job satisfaction are reciprocally related [48,50,51].

### The Purpose and Hypothesis of the Current Study

**Figure 1:**
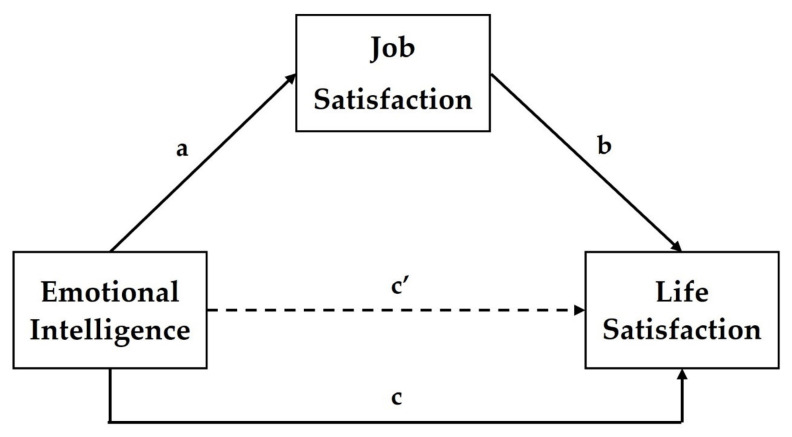
The hypothetical path model represents the mediating effect of job satisfaction on the relationship between emotional intelligence and life satisfaction; a, b, c, and c’ are path coefficients: a = the indirect effect of emotional intelligence (predictor) on job satisfaction as a mediator (H1); b = the indirect effect of job satisfaction (mediator) on life satisfaction as an explained variable (H2); c = the total effect of emotional intelligence (predictor) on life satisfaction as an explained variable (H3); c’ = the direct effect of emotional intelligence (predictor) on life satisfaction (explained variable) when job satisfaction (mediator) is included in the model of regression (H4).

The main aim of the present research is to examine the mediating role of job satisfaction on the relationship between emotional intelligence and life satisfaction among teachers during the COVID-19 pandemic (Figure 1). Although previous studies found associations between these three variables, the mediation model was never thoroughly tested. Since emotional intelligence is a positive predictor of job satisfaction [21,22,23,24,25,26,27,28,29] and life satisfaction [31,32,33,34,35,36,37,38,39,40,41,42,43], while job satisfaction is a strong positive predictor of life satisfaction [39,46,47,48,49,50,51,52,53], it is expected that emotional intelligence has an indirect positive impact on satisfaction with life, via job satisfaction. Brunetto et al. [29] found the partial mediating effect of well-being on the association between emotional intelligence and job satisfaction among police officers. However, since job satisfaction and life satisfaction (as a component of subjective well-being) are reciprocally related [48,50,51] the opposite direction between these variables is equally possible. Fu et al. [41] found the mediating effect of work engagement (strongly related to job satisfaction) on the relationship between emotional intelligence and general well-being in special education teachers from China. Finally, a recent study showed that trait emotion regulation directly affects life satisfaction, but there is no association between trait emotion regulation and intrinsic job satisfaction [40]. In contrast to this previous study [40], using a single component of emotional intelligence (trait emotion regulation) and job satisfaction (intrinsic dimension), this study will use a global composite score for the trait emotional intelligence and job satisfaction. The following hypotheses are formulated based on the above-presented literature review:

**Hypothesis** **1** **(H1).** *Emotional intelligence is a positive predictor of job satisfaction* (Figure 1, path a).

**Hypothesis** **2** **(H2).** *Job satisfaction is a positive predictor of life satisfaction* (Figure 1, path b).

**Hypothesis** **3** **(H3).** *There is a direct association between emotional intelligence and life satisfaction* (Figure 1, path c).

**Hypothesis** **4** **(H4).** *Emotional intelligence indirectly affects life satisfaction* (Figure 1, path c’) *via job satisfaction* (path a and path b).

## 2. Materials and Methods

This section describes the study design and procedure of the web-based survey among Polish teachers. The characteristics of the study sample and standardized questionnaires used to measure emotional intelligence, job satisfaction, and life satisfaction are presented in the next subsections. The statistical analysis used in the study to verify the hypotheses is introduced in the last subsection.

### 2.1. Study Design and Procedure

The cross-sectional study was performed between November 2020 and January 2021 during the second wave of the COVID-19 pandemic in Poland. A web-based survey (Google Forms) was disseminated to teachers using the snowball sampling method in two ways: (1) by e-mail (privately to friends’ teachers, *n* = 80); (2) by posting an invitation to participate in the research in Facebook groups for teachers (after obtaining permission from the group administrator). Here are the following groups: Innovative teacher, Creative teachers, Me, teacher, Teachers of grades 1–3 SP, Teachers’ market, Teacher in promotion, Polish language teachers, Music education, Everything for teachers, I teach casually, Genially-official Polish group, Microsoft Teams Poland.

The survey took about 15–20 min. The first web page of the survey included information on the research purpose and informed consent, and only those teachers who agreed could participate in the following parts of the study. The individuals were informed about anonymous and voluntary participation and the possibility of discontinuing the survey at any time. Several demographic questions were presented on the second webpage of the survey, including gender (Women, Men, I do not want to identify with any gender), age (years of age), education level (Primary, Junior high school, Vocational school, High school, College or university), work experience as a teacher (in years), teaching degree (Trainee teacher, Contract teacher, Appointed teacher, Certified teacher), a question regarding the current status of professional promotion, type of school (Primary school, High school, Technical secondary school, Trade school), subject taught by the teacher (e.g., early childhood education, foreign language, mathematics), the number of classes, the number of schools where the teacher is currently employed, and a question about the role of an educator. The standardized psychological questionnaire to measure life satisfaction, job satisfaction, and emotional intelligence was presented on the successive web pages of the online survey. The study protocol was approved by the University Research Ethics Committee at the University of Opole (No. 7/2021).

### 2.2. Participants

Initially, 332 people answered the invitation, but 10 of them refused to participate in the study. The study sample consisted of 322 teachers, aged 23–71 (*M* = 45.37, *SD* = 8.99), and the vast majority were women (*n* = 306, 95%), which reflects the typical structure of gender in education in Poland. Most of the teachers taught at primary school (*n* = 262, 81.36%), high school (*n* = 43, 13.35%), technical secondary school (*n* = 35, 10.87%), and in a trade school (*n* = 15, 4.65%). Among the respondents, 65% were class educators (*n* = 211), and the average work experience was 20 years (*M* = 20.68, *SD* = 10.32). The respondents had the following teaching degree: certified teacher (*n* = 217; 67.39%), appointed teacher (*n* = 57; 17.70%), contract teacher (*n* = 46; 14.28%), and trainee teacher (*n* = 2; 0.62%). Among the respondents, there were people in the process of professional promotion (*n* = 61; 18.94%). The respondents most frequently taught early childhood education (*n* = 79; 24.53%), a foreign language (*n* = 62; 19.25%), mathematics (*n* = 52; 16.14%), IT (*n* = 45; 13.97%), and Polish language (*n* = 40; 12.42%).

### 2.3. Measurement

#### 2.3.1. Emotional Intelligence

The Multi-Factor Emotional Intelligence Scale (MEIS) was developed by Schutte et al. [54] to examine the level of emotional intelligence following the theoretical model developed by Salovey and Mayer [55] which refers to the three key components: (1) expression, evaluation and perception of emotions; (2) practical use of emotions; (3) regulation of emotions. The MEIS scale was chosen because it can measure emotional intelligence understood as an ability (not a personality trait), and it has a reasonable number of questions assessing the global level of IE with high reliability. The self-report MEIS consists of 33 items with a five-point response scale (1 = *Strongly Disagree*, to 5 = *Strongly agree*). A total of 165 points can be obtained in the study, and higher scores indicate higher emotional intelligence. The internal reliability was 0.86 in the original study [54], and in the present sample Cronbach’s α = 0.97.

#### 2.3.2. Job Satisfaction

The Minnesota Satisfaction Questionnaire (MSQ) was used to assess job satisfaction among teachers [56]. The tool measures the degree to which vocational needs and values are satisfied on a job. The MSQ consists of 20 items with a standard five-point response scale (5 = *Very Satisfied*, 4 = *Satisfied*, 3 = *Neither Satisfied nor Dissatisfied*, 2 = *Dissatisfied*, and 1 = *Very Dissatisfied*). The MSQ provides in a short form the most comprehensive and valid assessment of general job satisfaction, including 20 facets, as well as intrinsic and extrinsic satisfaction with various aspects of work. Therefore, this measure was one of the most frequently used in the past decades and was selected for the present study purpose. Each item is representing one of the 20 facets of job satisfaction which are as follows: Ability Utilization, Achievement, Activity, Advancement, Authority, Company Policies and Practices, Compensation, Co-workers, Creativity, Independence, Moral Values, Recognition, Responsibility, Security, Social Service, Social Status, Supervision-Human Relation, Supervision-technical, Variety, and Working Conditions. The higher the score, the higher job satisfaction is presented. The internal reliability of the MSQ Cronbach’s α was between 0.85 and 0.91 in the original studies [56], while this study standardized Cronbach’s α = 0.93.

#### 2.3.3. Satisfaction with Life

The Satisfaction With Life Scale (SWLS) was developed by Diener et al. [44] to measure a global life satisfaction assessed cognitively rather than emotionally according to subjective criteria. This is a short form of a questionnaire (The first version consisted of 48 statements) that includes only five statements. A participant responds on a 7-point scale (from 1 = *Strongly Disagree* to 7 = *Strongly Agree*). The scores from all five items are summarized, and a higher composite score means greater life satisfaction. The results can be interpreted as 0–35 Extremely satisfied, 25–29 Satisfied, 20–24 Slightly satisfied, 15–19 Slightly dissatisfied, 10–14 Dissatisfied, and 5–9 Extremely dissatisfied [57]. This is the most frequently used and the best measure of the cognitive component of subjective well-being because it is a very short form linked to high reliability and validity. The original study found Cronbach’s α = 0.92 [47], while the present sample standardized Cronbach’s α was 0.89.

### 2.4. Statistical Analysis

The descriptive statistics were performed firstly to check the criteria for parametric tests. Kurtosis for emotional intelligence showed no appropriate values. Therefore, a non-parametric partial Spearman correlation was performed to examine the association between variables. Regression analysis was conducted for life satisfaction as an explained variable, job satisfaction and emotional intelligence as predictors, and age, gender, and job experience as confounding variables. The Durbin-Watson statistic was applied to check the assumption of autocorrelation, multicollinearity was examined using tolerance and Variance Inflation Factor (VIF), multivariate normality was tested using Shapiro-Wilk statistic, while heteroskedasticity was checked using Breusch-Pagan statistic. A multiple linear regression analysis (enter method) and mediation analysis were conducted to verify the hypotheses H1, H2, H3, and H4. The bias-corrected accelerated percentile bootstrap (BCa) based on 1000 replicates was used in the study to increase the accuracy of an estimate. Descriptive statistics, correlation, and regression analyses were performed using JASP software for Windows ver. 0.16.1, and mediation analysis was conducted using JAMOVI for Windows ver. 2.2.5.0.

## 3. Results

Descriptive statistics were performed initially to check the parametric properties of the variables (emotional intelligence, job satisfaction, and life satisfaction). Job satisfaction and life satisfaction showed good parametric properties, but kurtosis for emotional intelligence exceeded a range of scores ± 2 (see Table 1 for more details). Therefore, nonparametric analysis of partial Spearman correlation was used in the following analysis steps (Table 1). Emotional intelligence was related positively to life satisfaction and job satisfaction. Job satisfaction was also correlated positively with life satisfaction.

A multiple linear regression analysis was performed for life satisfaction as a dependent variable and job satisfaction and emotional intelligence as predictors. Since age, gender, and job experience were not related to dependent and independent variables, we decided not to include these variables in the model of regression. Age and job experience were strongly interrelated, which could assume multicollinearity. Initially, assumptions for the linear regression were examined. All statistics were suitable for the linear regression model, including the Durbin-Watson statistic (autocorrelation = −0.072, DW = 2.13, *p* = 0.230), tolerance (>0.3), VIF (<3), Shapiro-Wilk (*D* = 0.991, *p* = 0.058), and the Breusch-Pagan statistic (5.46, *p* = 0.065). Regression analysis showed that job satisfaction and emotional intelligence are positive predictors of life satisfaction (Table 2). The model explains for 30% of life satisfaction variance, *R*^2^ = 0.296, *F* (2, 319) = 67.14, *p* < 0.001.

**Figure 2:**
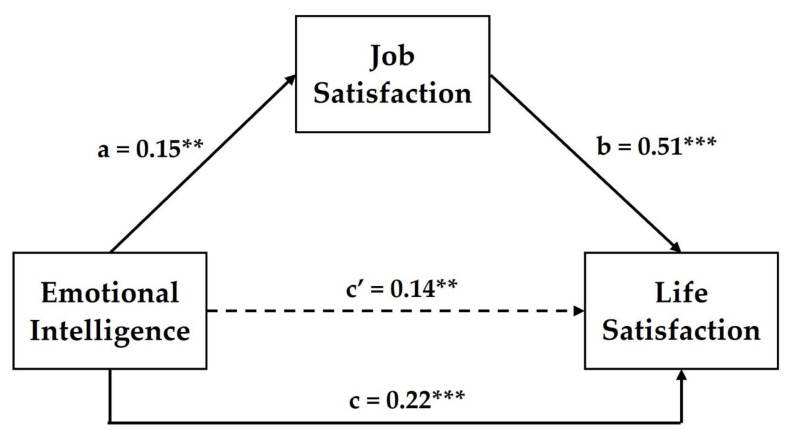
Path model of mediation; paths a and b represent the standardized regression weights for the indirect effect of emotional intelligence on life satisfaction via job satisfaction, while paths c and c’ are the standardized regression weights for total effect and direct effect, respectively. ** *p* < 0.01, *** *p* < 0.001.

Mediation analysis was conducted to examine the indirect effect of emotional intelligence on life satisfaction through job satisfaction. The results are presented in Table 3 and Figure 2. Emotional intelligence is a significant positive predictor of job satisfaction and life satisfaction, and job satisfaction is a significant positive predictor of life satisfaction. The total direct and indirect effects were significant, which indicates that job satisfaction partially mediates the relationship between emotional intelligence and life satisfaction.

## 4. Discussion

This study examined the mediating effect of job satisfaction on the relationship between emotional intelligence and life satisfaction among teachers from Poland during the second wave of the COVID-19 pandemic. For the first time, to the best of our knowledge, the mechanism of interplay between emotional intelligence and life satisfaction was explained partially by job satisfaction. As previously found [21,22,23,24,25,26,27,28,29], the research showed that emotional intelligence is a positive predictor of job satisfaction, so H1 was confirmed in this study. Also, life satisfaction can be predicted positively by job satisfaction (H2), which is consistent with the other studies [39,46,47,48,49,50,51,52,53]. This study proved that emotional intelligence and life satisfaction are interrelated, supporting H3 and previous studies [30,31,32,33,34,35,36,37,38,39,40,41,42,43]. Finally, H4 was confirmed indicating that job satisfaction partially plays a mediating role in associating emotional intelligence and life satisfaction. 

Subjective well-being is dependent on positive emotions and a favorable work environment, in which adult people spend a great part of their life. Numerous studies document the impact of emotional intelligence on satisfactory work, good physical and mental health, and well-being among teachers. Brunetto et al. [29] found that emotional intelligence leads to job satisfaction and well-being, with positive path relationships leading to employee engagement and organizational commitment. Sekreter [58] suggests that understanding students’ emotional state plays a key role in understanding their behavior and applying an ideal learning environment that encourages positive social interaction, active engagement, and a strong motivation to learn. A high teacher emotional competence contributes to the development of supportive teacher-student relationships, effective classroom management strategies, and successful emotional learning program implementation which in turn promote positive developmental outcomes in students’ achievements. Studies showed that high emotional intelligence is associated with high teachers’ classroom engagement [59,60] and contributes to an increase in student engagement [61] and student academic achievement [60]. Emotional intelligence plays a positive role in promoting students’ achievement by enhancing the effects of students’ self-perceptions of ability and self-esteem [62]. Studies found that emotional intelligence is a predictor of self-efficacy [60,63,64,65] and teacher effectiveness [20,61,63,65,66,67,68]. Furthermore, teachers’ age and experience were factors that impacted the association between emotional intelligence and organizational effectiveness in school circumstances [67].

Job satisfaction of the teachers has a significant effect on their life satisfaction, as was also found previously [46]. Job satisfaction is an essential domain of life satisfaction which has a beneficial impact on physical and mental health and a general sense of well-being. Job satisfaction depends on many factors, including financial satisfaction, full-time and permanent employment (compared to part-time and temporary work), occupational prestige, education, age, relationship status, and gender [53]. Also, an increase in work-life balance positively relates to life and work satisfaction [69]. However, an extensive survey study in 27 European countries indicates that job satisfaction increases subjective well-being, even controlling for working contract and welfare systems or inter-relations between work, family, and well-being [70]. 

Although all hypotheses were confirmed in the study, it is important to note that the model of mediation only partially explains just one-third of life satisfaction variance. This means that maybe some other variables are more effective predictors of satisfaction with life apart from job satisfaction and emotional intelligence. Therefore, more research is necessary to find the other factors of life satisfaction in teachers. For example, a more complex path model with self-efficacy, optimism as a trait, and the Big-five personality traits (neuroticism, extraversion, openness to experience, agreeableness, and conscientiousness), or some negative measures of mental health, such as stress, anxiety, depression, or burnout, could be examined in the future studies.

The study has several limitations that do not allow for generalization. Firstly, the convenience of snowball sampling and recruiting teachers to research online via social media is a limitation of this study. People who do not use social media and do not like teacher associations could not participate in the study. Secondly, self-report questionnaires may measure emotional intelligence with some bias. Future studies could use behavioral or physiological methods to assess emotional intelligence with greater accuracy. Although the teacher sample was quite large, it is not fully representative of the total population of teachers in Poland, particularly in other countries. Also, most of the participants were women, so future studies should be more balanced in gender and age. Finally, the structure of the sample can not be fully representative of the population of teachers in Poland. Cross-cultural research is necessary to verify the mediation model in the future. Also, longitudinal analysis is required since a cross-sectional design cannot allow for a causal association.

## 5. Conclusions

The study, for the first time (to the best of our knowledge), showed evidence of the mediating effect of job satisfaction on the relationship between emotional intelligence and life satisfaction among teachers. The study has several implications, in particular during such global crisis times as the COVID-19 pandemic. Emotional intelligence can be trained, so special prevention programs, enhancing emotional intelligence by improving skills of recognition, regulating, and managing emotions should be offered to teachers to increase their well-being during stressful work circumstances, such as a rapid transition from classroom face-to-face learning to distance online teaching. To improve job satisfaction among teachers, technical support, training in IT skills, and free access to applications that facilitate learning and increase the attractiveness of online classes should be offered in the school for teachers and students. Also, prevention and intervention programs should be focused on decreasing negative emotions, distress, anxiety, and depression to support the well-being of teachers and their students during the pandemic.

## Figures and Tables

**Table 1:** Descriptive statistics (*N* = 322).

Variable	Range	*M*	*SD*	Mdn.	Skewness	Kurtosis	Spearman’s Rho	
							2.	3.
1. Emotional Intelligence	41–165	121.65	23.59	126	−1.58	2.85		
2. Job Satisfaction	35–99	72.29	12.74	74	−0.5	0.06	0.21 ***	
3. Life Satisfaction	7–35	24.82	5.17	25	−0.61	0.57	0.31 ***	0.48 ***

*Note.* M = mean, SD = standard deviation, Mdn. = median. *** *p* < 0.001.

**Table 2:** Regression analysis for life satisfaction.

						95% BCa *CI*		Collinearity Statistics	
Variable	*B*	*SE B*	β	*t*	*p*	*LL*	*UL*	Tolerance	VIF
(Intercept)	6.24	1.75		3.56	<0.001	2.66	10.25		
Job Satisfaction	0.21	0.02	0.51	10.62	<0.001	0.16	0.25	0.98	1.02
Emotional Intelligence	0.03	0.01	0.14	2.99	0.003	0.00	0.05	0.98	1.02

*Note*. BCa = bias-corrected accelerated percentile bootstrap, *CI =* confidence interval, *LL* = lower level, *UL* = upper level, VIF = variance inflation factor.

**Table 3:** The mediating effects of job satisfaction on the association between emotional intelligence and life satisfaction.

				95% BCa *CI*				
Type	Effect	*b*	*SE*	*LL*	*UL*	β	*z*	*p*
Indirect	EI ⇒ JS ⇒ LS	0.017	0.007	0.004	0.032	0.076	2.26	0.024
Component	EI ⇒ JS	0.081	0.033	0.018	0.147	0.150	2.44	0.015
	JS ⇒ LS	0.205	0.022	0.162	0.251	0.505	9.19	<0.001
Direct	EI ⇒ LS	0.031	0.012	0.007	0.056	0.142	2.55	0.011
Total	EI ⇒ LS	0.048	0.012	0.024	0.071	0.218	4.00	<0.001

*Note.* EI = emotional intelligence, JS = job satisfaction, LS = life satisfaction, BCa = bias-corrected accelerated percentile bootstrap, *CI =* confidence interval, *LL* = lower level, *UL* = upper level. Delta method standard errors, ML estimator.

## Data Availability

Data supporting reported results can be found at Mendeley Data: Rogowska, A.; Meres, H. Emotional intelligence, job satisfaction and life satisfaction in Polish teachers during the second wave of the COVID-19 pandemic. Mendeley Data, 2022, doi: 10.17632/s2jkdcc3s5.1 Available online at https://data.mendeley.com/drafts/s2jkdcc3s5 (accessed on 1 June 2022).

## References

[1] UNESCO (2020). COVID-19 and Higher Education: Today and Tomorrow. Impact Analysis, Policy Responses and Recommendations.

[2] Colclasure B.C., Marlier A., Durham M.F., Brooks T.D., Kerr M. (2021). Identified challenges from faculty teaching at predominantly undergraduate institutions after the abrupt transition to emergency remote teaching during the COVID-19 pandemic. Educ. Sci..

[3] Daniel S.J. (2020). Education and the COVID-19 pandemic. Prospects.

[4] Marshall D.T., Shannon D.M., Love S.M. (2020). How teachers experienced the COVID-19 transition to remote instruction. Phi Delta Kappan.

[5] Neuwirth L.S., Jović S., Mukherji B.R. (2021). Reimagining higher education during and post-COVID-19: Challenges and opportunities. J. Adult Contin. Educ..

[6] Qazi A., Naseer K., Qazi J., Al Salman H., Naseem U., Yang S., Hardaker G., Gumaei A. (2020). Conventional to online education during COVID-19 pandemic: Do develop and underdeveloped nations cope alike. Child. Youth Serv. Rev..

[7] Bento F., Giglio Bottino A., Cerchiareto Pereira F., Forastieri de Almeida J., Gomes Rodrigues F. (2021). Resilience in Higher Education: A Complex Perspective to Lecturers’ Adaptive Processes in Response to the COVID-19 Pandemic. Educ. Sci..

[8] Jain S., Lall M., Singh A. (2021). Teachers’ voices on the impact of COVID-19 on school education: Are Ed-Tech companies really the panacea?. Contemp. Educ. Dialogue.

[9] Jelińska M., Paradowski M.B. (2021). Teachers’ perception of student coping with emergency remote instruction during the COVID-19 pandemic: The relative impact of educator demographics and professional adaptation and adjustment. Front. Psychol..

[10] Resnick B.A., Mui P.C., Bowie J., Kanchanaraksa S., Golub E., Sharfstein J.M. (2021). The COVID-19 pandemic: An opportunity to transform higher education in public health. Public Health Rep..

[11] Varalakshmi R., Arunachalam K. (2020). COVID 2019—Role of faculty members to keep mental activeness of students. Asian J. Psychiatr..

[12] Zhang X. Thoughts on large-scale long-distance web-based teaching in colleges and universities under novel coronavirus pneumonia epidemic: A case of Chengdu University. Proceedings of the 4th International Conference on Culture, Education and Economic Development of Modern Society (ICCESE 2020).

[13] Essary J.N., Barza L., Thurston R.J. (2020). Secondary traumatic stress among educators. Kappa Delta Pi Record.

[14] Slišković A., Maslić Seršić D. (2011). Work stress among university teachers: Gender and position differences. Arch. Hig. Rada Toksikol..

[15] Jakubowski T.D., Sitko-Dominik M.M. (2021). Teachers’ mental health during the first two waves of the COVID-19 pandemic in Poland. PLoS ONE.

[16] Ozamiz-Etxebarria N., Berasategi Santxo N., Idoiaga Mondragon N., Dosil Santamaría M. (2021). The psychological state of teachers during the COVID-19 crisis: The challenge of returning to face-to-face teaching. Front. Psychol..

[17] Watermeyer R., Crick T., Knight C., Goodall J. (2021). COVID-19 and digital disruption in UK universities: Afflictions and affordances of emergency online migration. High Educ..

[18] García-González M.A., Torrano F., García-González G. (2020). Analysis of Stress Factors for Female Professors at Online Universities. Int. J. Environ. Res. Public Health.

[19] Landolfi A., Barattucci M., De Rosa A., Lo Presti A. (2021). The Association of Job and Family Resources and Demands with Life Satisfaction through Work-Family Balance: A Longitudinal Study among Italian Schoolteachers during the COVID-19 Pandemic. Behav. Sci..

[20] Kumar A.C., Bhargavi S., Ravi P., Rohith S. (2021). The impact of emotional intelligence on teaching effectiveness and research quality of university faculty in India. NVEO.

[21] Anari N.N. (2012). Teachers: Emotional intelligence, job satisfaction, and organizational Commitment. J. Workplace Learnin..

[22] Kassim S.I., Bambale A.J., Jakada B.A. (2016). Emotional Intelligence and Job Satisfaction among Lecturers of Universities in Kano State: Empirical Evidence. J. Educ. Pract..

[23] Suleman Q., Syed M.A., Mahmood Z., Hussain I. (2020). Correlating emotional intelligence with job satisfaction: Evidence from a cross-sectional study among secondary school heads in Khyber Pakhtunkhwa, Pakistan. Front. Psychol..

[24] Güleryüz G., Güney S., Aydin E.M., Aşan O. (2008). The mediating effect of job satisfaction between emotional intelligence and organizational commitment of nurses: A questionnaire survey. Int. J. Nurs. Stud..

[25] Rajput S., Kaurav R.P.S., Ghanghoriya R. Do emotional intelligence always affect job satisfaction?. Proceedings of the 10th International Conference on Digital Strategies for Organizational Success.

[26] Sener E., Demirel O., Sarlak K. (2009). The effect of the emotional intelligence on job satisfaction. Stud. Health Technol. Inform..

[27] Lee H.J. (2018). How emotional intelligence relates to job satisfaction and burnout in public service jobs. Rev. Int. Des Sci. Adm..

[28] Munyug T.E., Ki Y.P., Fung C.Y. (2020). The relationship between emotional intelligence and job satisfaction among state civil servants. Int. J. Serv. Manag. Sust..

[29] Brunetto Y., Teo S.T.T., Shacklock K., Farr-Wharton R. (2012). Emotional intelligence, job satisfaction, well-being and engagement: Explaining organisational commitment and turnover intentions in policing. Hum. Res. Manag. J..

[30] Akinwumi O.A., Ayorinde M.O., Michael A.O. (2020). Emotional intelligence and job satisfaction as predictors of organizational commitment among private school teachers. GSJ.

[31] Guasp Coll M., Navarro-Mateu D., Giménez-Espert M.D.C., Prado-Gascó V.J. (2020). Emotional intelligence, empathy, self-esteem, and life satisfaction in spanish adolescents: Regression vs. QCA models. Front. Psychol..

[32] Afolabi O.A., Balogun A.G. (2017). Impacts of psychological security, emotional intelligence and self-efficacy on undergraduates’ life satisfaction. Psychol. Thought..

[33] Ain N.U., Munir M., Suneel I. (2021). Role of emotional intelligence and grit in life satisfaction. Heliyon.

[34] Cazan A.M., Năstasă L.E. (2015). Emotional Intelligence, Satisfaction with Life and Burnout among University Students. Procedia-Soc. Behav. Sci..

[35] Costa H., Ripoll P., Sánchez M., Carvalho C. (2013). Emotional intelligence and self-efficacy: Effects on psychological well-being in college students. Span. J. Psychol..

[36] Ruiz-Aranda D., Extremera N., Pineda-Galán C. (2014). Emotional intelligence, life satisfaction and subjective happiness in female student health professionals: The mediating effect of perceived stress. J. Psychiatr. Ment. Health Nurs..

[37] Runcan P.L., Iovu M. (2013). Emotional intelligence and life satisfaction in Romanian university students: The mediating role of self-esteem and social support. Rev. de Cercet si Interv Soc..

[38] Szczygieł D., Mikolajczak M. (2017). Why are people high in emotional intelligence happier? They make the most of their positive emotions. Pers. Individ. Differ..

[39] Augusto Landa J.M., López-Zafra E., Martínez de Antoñana R., Pulido M. (2006). Perceived emotional intelligence and life satisfaction among university teachers. Psicothema.

[40] Luque-Reca O., García-Martínez I., Pulido-Martos M., Lorenzo Burguera J., Augusto-Landa J.M. (2022). Teachers’ life satisfaction: A structural equation model analyzing the role of trait emotion regulation, intrinsic job satisfaction and affect. Teach. Teach. Educ..

[41] Fu W., Wang C., Tang W., Lu S., Wang Y. (2021). Emotional intelligence and well-being of special education teachers in china: The mediating role of work-engagement. Front. Psychol..

[42] Kong F., Gong X., Sajjad S., Yang K., Zhao J. (2019). How is emotional intelligence linked to life satisfaction? The mediating role of social support, positive affect and negative affect. J. Happiness Stud..

[43] Palmer B., Donaldson C., Stough C. (2002). Emotional intelligence and life satisfaction. Pers. Individ. Differ..

[44] Diener E., Emmons R.A., Larsen R.J., Griffin S. (1985). The Satisfaction with Life Scale. J. Pers. Assess..

[45] Alam F., Yang Q., Bhutto M.Y., Akhtar N. (2021). The influence of e-learning and emotional intelligence on psychological intentions: Study of stranded Pakistani students. Front. Psychol..

[46] Aydintan B., Koç H. (2016). The relationship between job satisfaction and life satisfaction: An empirical study on teachers. Int. J. Bus. Soc. Sci..

[47] Unanue W., Gómez M.E., Cortez D., Oyanedel J.C., Mendiburo-Seguel A. (2017). Revisiting the link between job satisfaction and life satisfaction: The role of basic psychological needs. Front. Psychol..

[48] Mishra V., Nielsen I., Smyth R., Newman A. (2014). The Job Satisfaction-Life Satisfaction Relationship Revisited: Using the Lewbel Estimation Technique to Estimate Causal Effects Using Cross-Sectional data.

[49] Coad A., Binder M. (2014). Causal linkages between work and life satisfaction and their determinants in a structural VAR approach. Econ. Lett..

[50] Bialowolski P., Weziak-Bialowolska D. (2021). Longitudinal Evidence for Reciprocal Effects Between Life Satisfaction and Job Satisfaction. J. Happiness Stud..

[51] Judge T.A., Watanabe S. (1993). Another look at the job satisfaction-life satisfaction relationship. J. Appl. Psychol..

[52] Keon T.L., McDonald B. (1982). Job satisfaction and life satisfaction: An empirical evaluation of their interrelationship. Hum. Relat..

[53] European Commission Directorate-General for Employment, Social Affairs and Inclusion (2016). Job Satisfaction and Satisfaction in Financial Situation and Their Impact on Life Satisfaction. https://ec.europa.eu/social/BlobServlet?docId=17504&langId=en.

[54] Schutte N.S., Malouff J.M., Hall L.E., Haggerty D.J., Cooper J.T., Golden C.J., Dornheim L. (1998). Development and validation of a measure of emotional intelligence. Pers. Individ. Differ..

[55] Salovey P., Mayer J.D. (1990). Emotional intelligence. Imag. Cogn. Pers..

[56] Weiss D.J., Dawis R.V., England G.W. (1967). Manual for the Minnesota Satisfaction Questionnaire. Minnesota Stud. Vocat. Rehab..

[57] Pavot W.G., Diener E., Colvin C.R., Sandvik E. (1991). Further validation of the Satisfaction with Life Scale: Evidence for the cross-method convergence of well-being measures. J. Pers. Assess..

[58] Sekreter G. (2019). Emotional intelligence as a vital indicator of teacher effectiveness. Int. J. Soc. Sci. Educ. Stud..

[59] Abiodullah M., Sameen D., Aslam M. (2020). Emotional intelligence as a predictor of teacher engagement in classroom. Bull. Educ. Res..

[60] Wang L. (2022). Exploring the relationship among teacher emotional intelligence, work engagement, teacher self-efficacy, and student academic achievement: A moderated mediation model. Front. Psychol..

[61] Chauhan N., Bandi R.S. (2021). Role of emotional intelligence on teaching effectiveness and student engagement- a contextual framework. JETIR.

[62] Curci A., Lanciano T., Soleti E. (2014). Emotions in the classroom: The role of teachers’ emotional intelligence ability in predicting students’ achievement. Am. J. Psychol..

[63] Anwar R.H., Zaki S., Memon N., Thurasamy R. (2021). Exploring the interplay of trait emotional intelligence and ESL teacher effectiveness: Is self-efficacy the mechanism linking them?. SAGE Open.

[64] Mahasneh A.M. (2016). Emotional intelligence as a predictor of teacher sense of self-efficacy among student teachers in Jordan. N. Am. J. Psychol..

[65] Valente S., Lourenço A.A., Alves P., Domínguez-Lara S. (2020). The role of emotional intelligence capacity for teacher’s efficacy and classroom management efficacy. Rev. CES Psicol..

[66] Adeyemo D.A., Chukwudi A.R. (2014). Emotional intelligence and teacher efficacy as predictors of teacher effectiveness among pre-service teachers in some Nigerian universities. Int. J. Eval. Res. Educ..

[67] Jha A., Singh I. (2012). Teacher effectiveness in relation to emotional intelligence among Medical and Engineering Faculty members. Eur. J. Psychol..

[68] Soanes D., Sungoh S. (2019). Influence of emotional intelligence on teacher effectiveness of science teachers. Psychology.

[69] Žnidaršič J., Marič M. (2021). Relationships between work-family balance, job satisfaction, life satisfaction and work engagement among higher education lecturers. Organizacija.

[70] Cannas M., Sergi B.S., Sironi E., Mentel U. (2019). Job satisfaction and subjective well-being in Europe. Econ. Soc..

